# Clinical safety and efficacy of vitamin D3 analog ointment for treatment of obstructive meibomian gland dysfunction

**DOI:** 10.1186/s12886-017-0482-7

**Published:** 2017-06-07

**Authors:** Reiko Arita, Motoko Kawashima, Masataka Ito, Kazuo Tsubota

**Affiliations:** 1 Department of Ophthalmology, Itoh Clinic, 626-11 Minami-Nakano, Minumaku, Saitama, Saitama 337-0042 Japan; 2 Department of Ophthalmology, Keio University, Tokyo, Japan; 3 Department of Developmental Anatomy and Regenerative Biology, National Defense Medical College, Saitama, Japan

**Keywords:** Vitamin D3, Ointment, Meibomian gland dysfunction, Meibomian gland obstruction

## Abstract

**Background:**

Hyperkeratinization is a major cause of obstructive meibomian gland dysfunction (oMGD) and results in degenerative gland dilation and atrophy without inflammation. Ointment containing 1,25-dihydroxy-22-oxavitamin D3 (maxacalcitol), a noncalcemic analog of the active form of vitamin D3, is applied for the treatment of hyperkeratotic cutaneous conditions such as psoriasis and ichtyosis because it suppresses the proliferation and promotes the differentiation of keratinocytes through interaction with the vitamin D receptor. The aim of the present study was to evaluate the safety and efficacy of maxacalcitol ointment for the treatment of oMGD.

**Methods:**

Six eyes of six healthy male subjects (mean age ± SD, 36.4 ± 10.8 years) and 12 eyes of eight oMGD patients (five men and three women; mean age ± SD, 55.6 ± 13.2 years) were enrolled in the study. Maxacalcitol ointment was applied to the upper and lower lid margins twice a day for 8 weeks. Subjective symptoms, lid margin abnormalities, tear film breakup time (BUT), ocular surface staining, meibum grade, Schirmer test value, and meibomian gland area were evaluated in the oMGD patients before, during, and after the treatment period.

**Results:**

Severe adverse effects of ointment application were not observed in the healthy subjects or oMGD patients. The clinical scores for plugging of meibomian gland orifices and lid margin vascularity as well as BUT, meibum grade, and meibomian gland area were significantly improved in oMGD patients after the 8-week treatment period compared with pretreatment values (*P* values of <0.001, 0.020, 0.030, 0.020, and 0.017, respectively).

**Conclusions:**

Topical eyelid application of an analog of the active form of vitamin D3 was found to be safe as well as to improve the condition of patients with oMGD. Such ointment thus warrants further evaluation as a potential new treatment option for this condition.

**Trial registration:**

This study was registered with the UMIN database (ID: UMIN000016230) on 16 January 2015.

**Electronic supplementary material:**

The online version of this article (doi:10.1186/s12886-017-0482-7) contains supplementary material, which is available to authorized users.

## Background

Meibomian glands are specialized sebaceous glands that secrete the oily layer of the tear film, which prevents the evaporation of tear fluid. Meibomian gland dysfunction (MGD) is a chronic, diffuse abnormality of meibomian glands that is characterized by terminal duct obstruction or qualitative or quantitative changes in glandular secretion that result in thinning of the lipid layer of the tear film and consequent tear film instability [1]. MGD is a relatively common condition and yet is often overlooked in ophthalmic practice. Population-based studies have suggested that the prevalence of MGD is higher in Asians than in other ethnic groups, with reported values of 46.2% in Thailand [2], 60.8% in Taiwan [3], 61.9% in Japan [4], and 69.3% in China [5]. In addition, in one study, more than 80% of patients with dry eye were found to have MGD [6].

The International Workshop on Meibomian Gland Dysfunction recommended various options for the management and treatment of the various stages of MGD [7]. Such options include application of a warm compress [7, 8] or lipid-based tear substitutes [9], lid hygiene [7], oral or topical administration of anti-inflammatory agents (minocycline [10], tetracycline [11], or azithromycin [12]), insertion of an intraductal probe [13], and exposure to intense pulsed light [14].

Several factors including hyperkeratinization of the gland orifice [15], ocular surface desiccation [16], and a decline in meibocyte differentiation related to aging [17] have been implicated in the pathophysiology of MGD. In particular, hyperkeratinization is a major cause of obstructive MGD (oMGD) and is thought to result in the adherence of sloughed epithelial cells and gland blockage, cystic dilation, and atrophy [16] without inflammation [15]. Ointment containing 1,25-dihydroxy-22-oxavitamin D3 (maxacalcitol), a noncalcemic analog of the active form of vitamin D3, is used for the treatment of hyperkeratotic cutaneous conditions such as psoriasis, ichtyosis, and acne vulgaris [18] because it suppresses the proliferation and promotes the differentiation of keratinocytes through interaction with the vitamin D receptor [19, 20]. The application of maxacalcitol is thus a potential treatment option for oMGD. We have demonstrated the safety and efficacy of such ointment in an animal model of oMGD [21]. We have now examined the safety of maxacalcitol ointment in healthy subjects as well as its therapeutic efficacy in patients with oMGD.

## Methods

### Subjects

To examine the safety of maxacalcitol ointment for eyelid application, we enrolled six eyes of six healthy men (mean age ± SD, 36.4 ± 10.8 years) on the basis of the standard procedure that requires a small number of male participants for a phase I clinical trial. Data were obtained from the left eye of each healthy subject. The therapeutic efficacy of the ointment was evaluated with 12 eyes of eight patients (five men and three women; mean age ± SD, 55.6 ± 13.2 years) who were diagnosed with oMGD and showed no improvement after previous treatments. oMGD was diagnosed on the basis of previously described criteria [22]: (1) at least one symptom such as ocular fatigue, discharge, foreign body sensation, dryness, uncomfortable sensation, sticky sensation, pain, epiphora, itching, redness, heavy sensation, glare, excessive blinking, burning sensation, and ocular discomfort on arising; (2) at least one lid margin abnormality (details are described below); and (3) poor meibum secretion. Exclusion criteria for both healthy subjects and oMGD patients included ocular allergies, contact lens wear, continual eyedrop use, history of eye surgery, and systemic or other ocular diseases that might interfere with tear film production or function. Individuals whose eyes showed excessive meibum secretion were also excluded.

### Procedures

Maxacalcitol ointment (Oxarol; Maruho, Osaka, Japan) was applied twice a day for 8 weeks to the upper and lower lid margins of both healthy subjects and oMGD patients. Examinations were performed before as well as 4 and 8 weeks after the onset of treatment. The examinations were performed sequentially as follows: (1) Subjects were questioned regarding the absence or presence of 14 ocular symptoms (ocular fatigue, discharge, foreign body sensation, dryness, uncomfortable sensation, sticky sensation, pain, epiphora, itching, redness, heavy sensation, glare, excessive blinking, and burning sensation) [23], which were then scored from 0 to 14 according to the number present. (2) Four abnormalities of the upper and lower lid margins (vascularity, plugging, irregularity, and displacement of the mucocutaneous junction) were graded, with lid margin vascularity and plugging of meibomian gland orifices being graded from 0 to 3 and irregularity of lid margins being graded from 0 to 2 (Table 1) [24]. Displacement of the mucocutaneous junction (MCJ) was scored as 0 (Marx’s line is entirely on the conjunctival side of meibomian gland orifices), 1 (Marx’s line is in contact with some gland orifices), 2 (Marx’s line courses through all gland orifices), or 3 (Marx’s line is entirely on the eyelid margin side of the gland orifices) [25]. The scores for the upper and lower eyelids were averaged for each abnormality. (3) Fluorescein staining of the ocular surface was divided into three zones comprising nasal conjunctival, corneal, and temporal conjunctival areas [26]. The staining score ranged from 0 to 3 for each zone, yielding a total superficial punctate keratopathy (SPK) score of 0 to 9 for the ocular surface. (4) Tear film breakup time (BUT) was measured after instillation of 1 μl of a preservative-free solution of 1% fluorescein dye into the conjunctival sac with the use of a micropipette and the subjects were asked to blink several times. BUT was measured three times consecutively with a stopwatch, and the mean of the three values was calculated. (5) The upper and lower eyelids were examined with the use of a noncontact meibography system (SL-D701 DC4 BG-5; Topcon, Tokyo, Japan), and partial or complete loss of meibomian glands was scored according to the meiboscore for each eyelid as previously described [27]. Meibomian gland area was calculated by software developed in-house [28]. (6) A Schirmer strip (Whatman no. 41; Showa, Tokyo, Japan) was inserted over the lower lid margin (midway between the middle and outer thirds) for 5 min without topical anesthesia. Subjects were asked to close their eyes during the measurement. (7) Digital pressure was applied to the upper tarsus, and the degree of ease with which meibum release was induced was evaluated semiquantitatively on a scale of 0 to 3 [4].

**Table 1:** Abnormal lid margin findings

Lid margin vascularity	
0 = No or slight redness of lid margin conjunctiva and no telangiectasia crossing meibomian gland orifices	
1 = Redness of lid margin conjunctiva and no telangiectasia crossing meibomian gland orifices	
2 = Redness of lid margin conjunctiva and telangiectasia crossing meibomian gland orifices with a distribution of less than half of the full length of the lid	
3 = Redness of lid margin conjunctiva and telangiectasia crossing meibomian gland orifices with a distribution of half or more of the full length of the lid	
Plugging of meibomian gland orifices	
0 = No plugging of gland orifices	
1 = Plugging of fewer than three gland orifices	
2 = Plugging of three or more gland orifices with a distribution of less than half of the full length of the lid	
3 = Plugging of three or more gland orifices with a distribution of half or more of the full length of the lid	
Lid margin irregularity	
0 = No lid margin irregularity	
1 = Fewer than three lid margin irregularities with shallow notching	
2 = Three or more lid margin irregularities with shallow notching or any irregularities with deep notching	

### Statistical analysis

Quantitative data are presented as means ± SD. Parameters were compared between before and either during or after treatment with the use of one-way ANOVA (Dunnett’s test). A *P* value of <0.05 was considered statistically significant.

## Results

### Safety of maxacalcitol ointment for eyelid application

One of the six normal subjects showed eyelid hyperemia at the area of ointment application, which was thought to be contact dermatitis, on the 3rd day of the treatment regimen. No adverse effects including ocular symptoms such as redness, pain, or chemosis were detected in the other healthy subjects throughout the 8-week treatment period.

### Efficacy of maxacalcitol ointment in patients with oMGD

Application of maxacalcitol ointment for 4 weeks was associated with a significant improvement in ocular symptom score and lid margin vascularity compared with before treatment (Table 2). Furthermore, ointment application for 8 weeks was associated with a significant improvement in ocular symptom score, plugging of meibomian gland orifices, lid margin vascularity, tear film BUT, SPK score, meibum grade, and meibomian gland area compared with before treatment (Table 2). Detailed information of each subject was described in the Additional file 1 (Detailed data of the subjects at pre-, 1 months after and 2 months after treatment with Vit D3).

**Table 2:** Changes in clinical parameters for oMGD patients between before and 4 or 8 weeks after the onset of maxacalcitol treatment

Parameter	Pretreatment	4 weeks	8 weeks
Ocular symptoms	6.9 ± 2.5	4.0 ± 1.2 (0.001)	2.2 ± 1.3 (<0.001)
Plugging of gland orifices (0–3)	1.8 ± 0.8	0.9 ± 0.7 (0.070)	0.4 ± 0.5 (<0.001)
Lid margin irregularity (0–2)	1.0 ± 0.6	0.8 ± 0.4 (0.597)	0.8 ± 0.4 (0.597)
Lid margin vascularity (0–3)	1.3 ± 0.5	0.6 ± 0.5 (0.020)	0.6 ± 0.5 (0.020)
MCJ replacement (0–3)	1.0 ± 0.6	0.8 ± 0.4 (0.633)	0.7 ± 0.5 (0.197)
BUT (s)	2.9 ± 1.4	4.2 ± 1.7 (0.163)	5.4 ± 2.2 (0.030)
SPK score (0–9)	0.5 ± 0.5	0.3 ± 0.5 (0.519)	0.5 ± 0.0 (0.011)
Meibum grade (0–3)	2.0 ± 1.0	1.3 ± 0.8 (0.105)	0.8 ± 0.8 (0.020)
Schirmer test value (mm)	6.5 ± 1.1	6.9 ± 1.0 (0.548)	6.6 ± 1.1 (0.974)
Mean meibomian gland area (%)	24.0 ± 8.3	27.7 ± 7.3 (0.399)	32.8 ± 7.5 (0.017)

Values in parentheses are *P* values for comparison with the corresponding pretreatment data (one-way ANOVA followed by Dunnett’s test)

### Case presentation

**Fig. 1:**
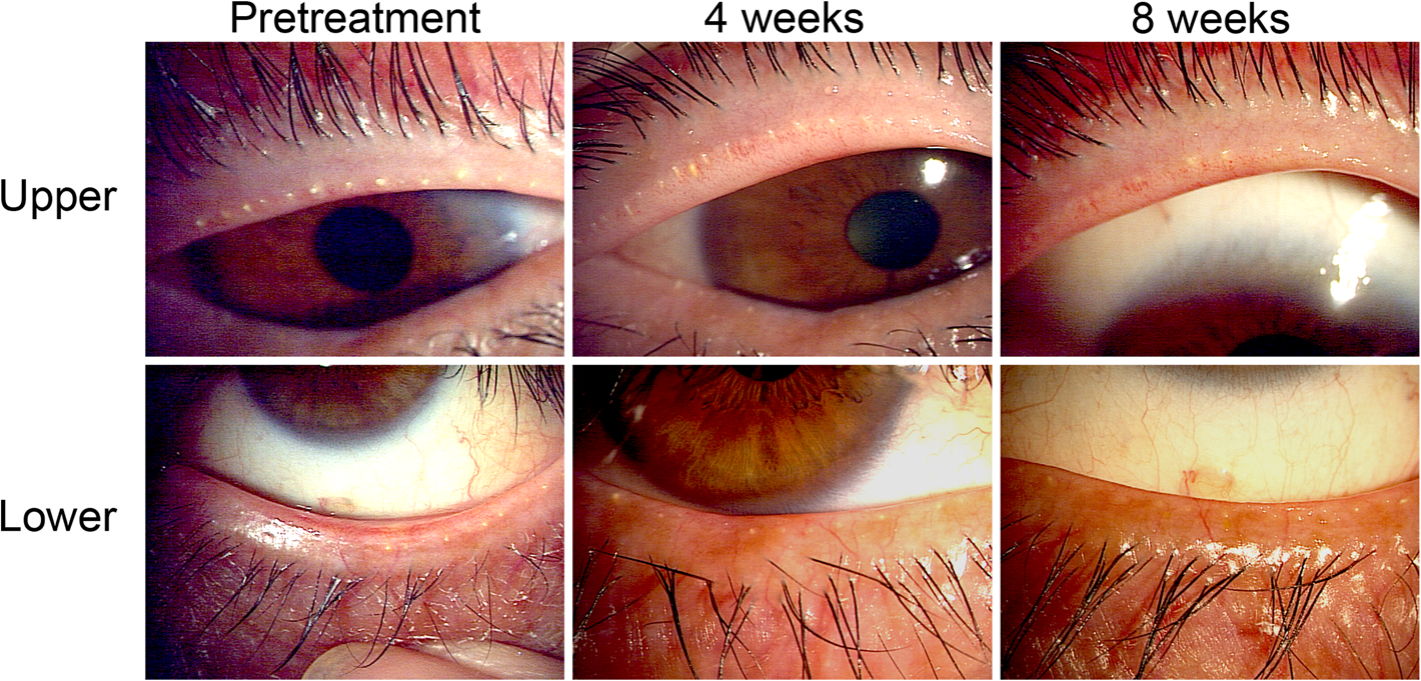
Lid margin abnormalities in a 65-year-old man with oMGD. The right eye of a 65-year-old man with oMGD is shown before as well as 4 and 8 weeks after the onset of treatment with maxacalcitol ointment. Note that plugging of meibomian gland orifices as well as lid margin vascularity were ameliorated after treatment for 8 weeks

**Fig. 2:**
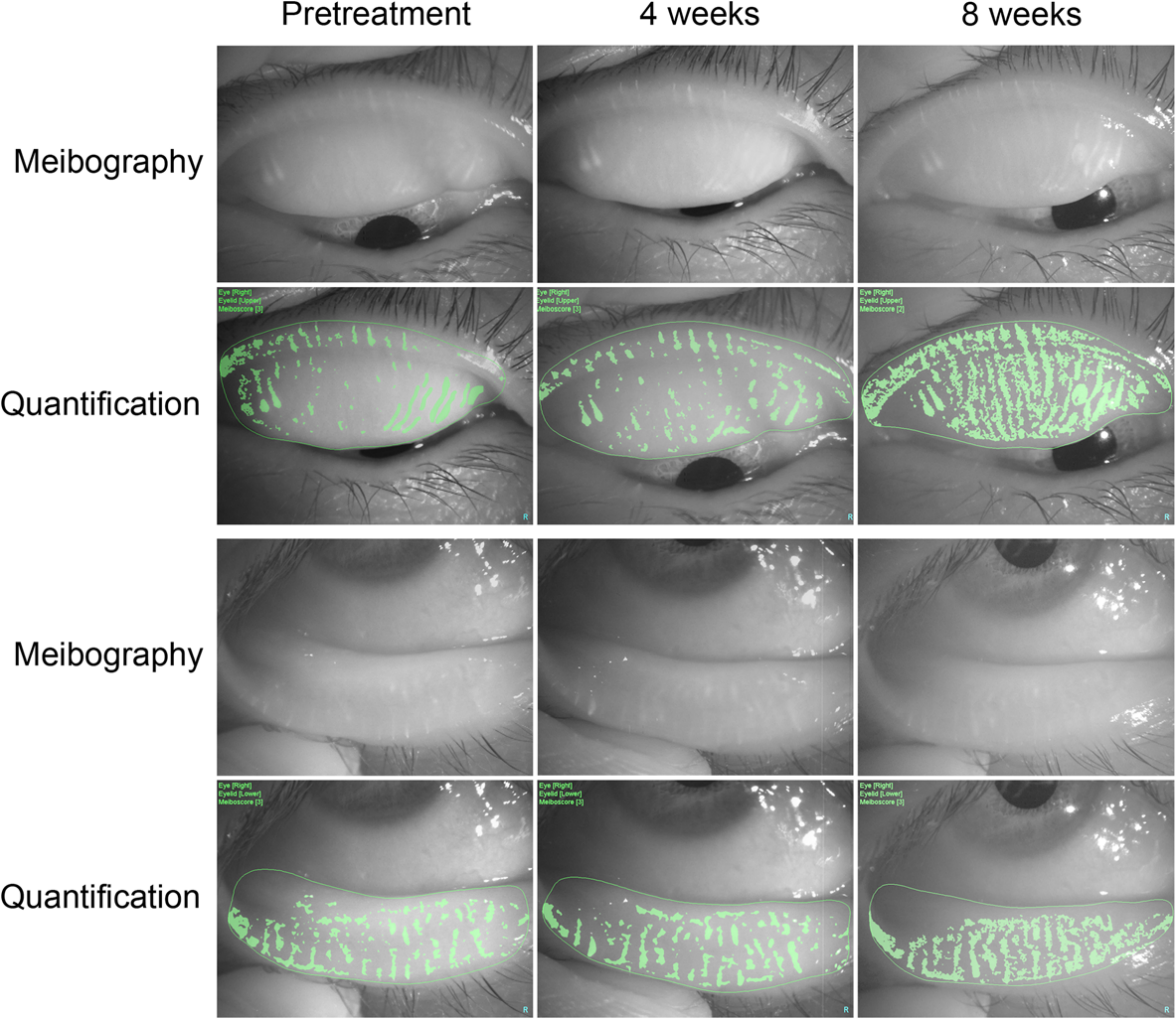
Meibomian gland area for the same patient as in Fig. 1. Regions shown in *green* represent meibomian glands and dilated gland ducts as revealed by noninvasive meibography and analysis software. Meibomian gland area in the upper eyelid (upper two panels) was 11.9% before treatment, 12.4% after treatment for 4 weeks, and 36.3% after treatment for 8 weeks. The corresponding values for the lower eyelid (bottom two panels) were 16.6% before treatment, 15.8% after treatment for 4 weeks, and 26.5% after treatment for 8 weeks

A 65-year-old man in the oMGD group of subjects had severe ocular discomfort before maxacalcitol ointment application. Plugging of meibomian gland orifices and lid margin vascularity were both graded 3, tear film BUT was 3 s, meibum grade was 3, the Schirmer test value was 14 mm, and mean meibomian gland area was 14.3%. The patient showed no corneal or conjunctival staining with fluorescein. Four weeks after the onset of ointment application, both plugging of gland orifices and lid margin vascularity were slightly improved, whereas meibomian gland area was not obviously changed (Figs. 1 and 2). After treatment for 8 weeks, plugging of gland orifices (grade 2), lid margin vascularity (grade 2), BUT (6 s), meibum grade (grade 1), and mean meibomian gland area (31.4%) were all improved. The patient manifested no corneal or conjunctival staining and the Schirmer test value remained unchanged after the 8 weeks of treatment.

## Discussion

We have here demonstrated the safety and efficacy of ointment containing an analog of the active form of vitamin D3 (maxacalcitol) in healthy subjects and oMGD patients, respectively. Application of the ointment for 8 weeks was thus associated with a significant improvement in clinical parameters in oMGD patients, suggesting that it might provide a new treatment option for this condition.

The physiological effects of the active form of vitamin D3 include inhibition of both hyperkeratinization and inflammation [29, 30]. Obstruction of meibomian gland orifices in many individuals with oMGD is due to hyperkeratinization around the gland orifices. We have now shown that the application of maxacalcitol ointment suppressed hyperkeratinization around meibomian gland orifices in oMGD patients and that this effect was accompanied by increased outflow of meibum and improvement in tear film parameters. In addition, the anti-inflammatory effect of maxacalcitol may directly ameliorate the chronic inflammation that can be associated with oMGD as a result of stagnation of meibum in meibomian glands. This dual action of maxacalcitol targeting both hyperkeratinization and inflammation may thus contribute to the observed improvement in clinical parameters related to oMGD.

The application of maxacalcitol ointment was also associated with an increase in the area of meibomian glands including dilated gland ducts in oMGD patients, as revealed by noninvasive meibography [27] and analysis with our in-house software [28]. As far as we are aware, the possible effects of the active form of vitamin D3 on acinar cells or ductal cells of meibomian glands have not been investigated. Our results suggest that obstruction of meibomian glands may suppress meibum production, and that the release of such obstruction allows acinar cells to resume stable production of meibum and increases meibomian gland area.

In contrast, lid margin irregularity, MCJ replacement, and the Schirmer test value in oMGD patients were not affected by application of maxacalcitol ointment. Lid margin irregularity is attributable to the dropout of meibomian glands, which is thought to reflect gland atrophy or fibrosis. The inhibitory effects of the active form of vitamin D3 on hyperkeratinization and inflammation would thus not be expected to have an impact on lid margin irregularity. The lack of change in Schirmer test value also may indicate that the active form of vitamin D3 does not affect (positively or negatively) lacrimal glands. Furthermore, the improvement in tear film BUT and SPK score observed in oMGD patients after treatment with maxacalcitol might have resulted at least in part from the oily nature of the applied ointment.

Our results suggest that analogs of the active form of vitamin D3 may be widely applicable to the treatment of oMGD. The International Workshop on Meibomian Gland Dysfunction [7] suggested that the first line of MGD treatment should be to provide patients with information on their condition, with implementation of lid hygiene together with eye warming and the administration of eyedrops for dry eye and topical lubricants being recommended as second-line treatment. A third line of treatment would be eye ointments and oral administration of tetracycline, and a fourth line would be anti-inflammatory therapy for dry eye. We have now shown that the anti-hyperkeratinization action of maxacalcitol ointment is likely responsible for its attenuation of the plugging of meibomian gland orifices in oMGD patients. Given that the aim of implementation of lid hygiene is to clean around and thereby to unplug gland orifices, topical application of analogs of the active form of vitamin D3 may also constitute a second line of MGD treatment. It may thus constitute an active form of lid hygiene by resulting not only in the physical removal of waste from around the gland orifices but also in an improvement in the condition of the area around the orifices.

One of the six healthy subjects in the present study developed lid hyperemia at the site of ointment application, suggestive of contact dermatitis. The frequency of contact dermatitis associated with application of maxacalcitol to skin was found to be 0.48% (interview form for Oxarol) [31]. It is not known whether the frequency for the eyelid differs from that for other parts of the skin. Discontinuation of ointment application to skin resulted in rapid improvement in symptoms, as was found to be the case for the affected healthy subject in the present study. The occurrence of contact dermatitis due to topical treatment with the active form of vitamin D3 thus appears to be infrequent, with the condition being mild and reversible. Such treatment may thus be acceptable for the eyelid in patients with oMGD.

Our study has several limitations. First, this trial of maxacalcitol ointment included only a single arm. Definitive demonstration of the efficacy of maxacalcitol for treatment of oMGD will require the performance of a double-blind clinical study. Second, the observation period of 8 weeks was not long enough to reveal long-term efficacy for this chronic disease, although our results are highly suggestive of short-term efficacy. Third, our method for evaluation of meibomian gland area by meibography is based on two-dimensional images and thus does not account for gland volume. Further improvement of the software may resolve this limitation.

## Conclusions

We found that ocular symptoms, plugging of meibomian gland orifices, lid margin vascularity, tear film BUT, SPK score, meibum grade, and meibomian gland area were all significantly improved in oMGD patients after application of maxacalcitol ointment for 8 weeks compared with pretreatment values. Our results thus suggest that analogs of the active form of vitamin D3 may be effective for the treatment of oMGD and that they warrant further investigation in clinical trials with larger numbers of patients.

## Additional file

Additional file 1: Detailed data of the subjects at pre-, one months after and two months after treatment with Vit D3. (XLSX 18 kb)

## Abbreviations

BUT: Breakup time
MCJ: Mucocutaneous junction
oMGD: Obstructive meibomian gland dysfunction
SPK: Superficial punctate keratopathy
